# Associations of dietary patterns between age 9 and 24 months with risk of celiac disease autoimmunity and celiac disease among children at increased risk

**DOI:** 10.1016/j.ajcnut.2023.08.009

**Published:** 2023-10-16

**Authors:** Elin M. Hård af Segerstad, Lazarus K. Mramba, Xiang Liu, Ulla Uusitalo, Jimin Yang, Jill Norris, Suvi M. Virtanen, Edwin Liu, Kalle Kurppa, Sibylle Koletzko, Annette G. Ziegler, Jorma Toppari, Marian Rewers, Beena Akolkar, Jeffrey P. Krischer, Carin Andrén Aronsson, Daniel Agardh, for the TEDDY study group

**Affiliations:** 1 Department of Clinical Sciences, Lund University, Malmö, Sweden; 2 Department of Pediatrics, Health Informatics Institute, Morsani Collage of Medicine, University of South Florida, Tampa, FL, United States; 3 Department of Epidemiology, Colorado School of Public Health, University of Colorado, Aurora, CO, United States; 4 Department of Public Health and Welfare, Finnish Institute for Health and Welfare, Helsinki, Finland; 5 Unit of Health Sciences, Faculty of Social Sciences, Tampere University, Tampere, Finland; 6 Tampere Center for Child, Adolescent and Maternal Health Research, Tampere University and Tampere University Hospital, Tampere, Finland; 7 Children’s Hospital Colorado, University of Colorado Denver, Aurora, CO, United States; 8 Faculty of Medicine and Health Technology, Tampere University, Tampere, Finland; 9 The University Consortium of Seinäjoki, Seinäjoki, Finland; 10 Department of Paediatrics, Dr. von Hauner Children’s Hospital, University Hospital, LMU Munich, Munich, Germany; 11 Department of Paediatrics, Gastroenterology and Nutrition, School of Medicine Collegium Medicum University of Warmia and Mazury, Olsztyn, Poland; 12 Klinikum Rechts der Isar, Technische Universität München, München, Bayern, Germany; 13 Institute of Diabetes Research, Helmholtz Zentrum München, Germany; 14 Forschergruppe Diabetes e.V, Neuherberg, Germany; 15 Research Centre for Integrative Physiology and Pharmacology, Institute of Biomedicine, University of Turku, Turku, Finland; 16 Department of Paediatrics, Turku University Hospital, Turku, Finland; 17 Barbara Davis Center for Childhood Diabetes, University of Colorado, Aurora, CO, United States; 18 National Institute of Diabetes and Digestive and Kidney Disease, National Institutes of Health, Bethesda, MD, United States

**Keywords:** celiac disease autoimmunity, celiac disease, complementary feeding, dietary patterns, gluten, HLA-DQ2/DQ8, infant diet, principal component analysis, TEDDY

## Abstract

**Background:**

Higher gluten intake in childhood is associated with increased incidence of celiac disease autoimmunity (CDA) and celiac disease. It remains to be studied whether different dietary patterns independent of gluten intake contribute to the incidence.

**Objectives:**

This study aimed to explore associations of dietary patterns by age 2 y with risk of CDA and celiac disease in genetically susceptible children.

**Methods:**

Data was used from 6726 participants at genetic risk of type 1 diabetes and celiac disease enrolled in the observational cohort, The Environmental Determinants of Diabetes in the Young (TEDDY) study. Children were annually screened for tissue transglutaminase autoantibodies (tTGAs) from age 2 y. Principal component analysis extracted dietary patterns, based on intake of 27 food groups assessed by 3-d food records at age 9 to 24 mo. The primary outcome was CDA (i.e., persistently tTGA-positive in at least 2 consecutive samples), and the secondary outcome was celiac disease. During follow-up to mean age 11.0 (standard deviation 3.6) y, 1296 (19.3%) children developed CDA, and 529 (7.9%) were diagnosed with celiac disease. Associations of adherence to dietary patterns (per 5-unit increase) with the study outcomes were estimated by Cox regression models adjusted for risk factors including gluten intake.

**Results:**

At age 9 mo, a dietary pattern higher in the food groups vegetable fats and milk was associated with reduced risk of CDA (hazard ratio [HR]: 0.88; 95% confidence interval [CI]: 0.79, 0.98; *P* = 0.02). At 24 mo, a dietary pattern higher in the food groups wheat, vegetable fats, and juices, and lower in milk, meat, and oats at age 24 mo was associated with increased risk of CDA (HR: 1.18; 95% CI: 1.05, 1.33; *P* < 0.001) and celiac disease (HR: 1.24; 95% CI: 1.03, 1.50; *P* = 0.03).

**Conclusions:**

Dietary patterns in early childhood are associated with risk of CDA and celiac disease in genetically predisposed children, independent of gluten intake.

## Introduction

Celiac disease is a chronic immune-mediated disease caused by intolerance to dietary gluten and is strongly associated with the human leukocyte antigen (HLA)-DQ2 and DQ8 haplotypes [1]. However, genetics is estimated to explain only approximately half of the disease risk, indicating that environmental exposures may serve as additional triggers [1]. A higher gluten intake in early life has been associated with increased incidence of celiac disease autoimmunity (CDA) and celiac disease in children at genetic risk in some [[2], [3], [4], [5]] but not all prospective birth cohorts [6]. However, genetics and amount of gluten intake only partly explains the observed differences in incidence between countries [2,7].

Investigating the relationship between dietary patterns and health outcomes allows for capturing full dietary exposure closer to true intake instead of studying single foods or nutrients [8]. Several methods to investigate dietary patterns are used in nutritional research, either by a hypothesis-driven or exploratory approach, of which principal component analysis (PCA) is one of the most commonly applied methods. PCA is a data-driven method that finds dietary patterns that are present in the study population, unrestricted by the investigator’s à priori hypothesis [9].

A western diet, characterized by high intake of saturated fats, sugar, and ultraprocessed foods and low intake of fiber, has been associated with the increased risk of allergic and chronic inflammatory conditions [[10], [11], [12], [13]] as well as with higher levels of proinflammatory biomarkers [14]. More recently, a prudent dietary pattern high in vegetables, potatoes, pasta, and rice, and low in refined cereals and sweetened beverages, was recently associated with the reduced risk of CDA in a small study on children [15]. However, this finding has not been validated in other prospective cohorts, and the impact of childhood dietary exposure besides the effect of gluten is yet to be studied.

The aim of the present study was to explore associations of early life dietary patterns independent of gluten intake between 9 and 24 mo of age with risk of CDA and celiac disease in genetically at-risk children prospectively followed from birth.

## Methods

### Study population

**FIGURE 1:**
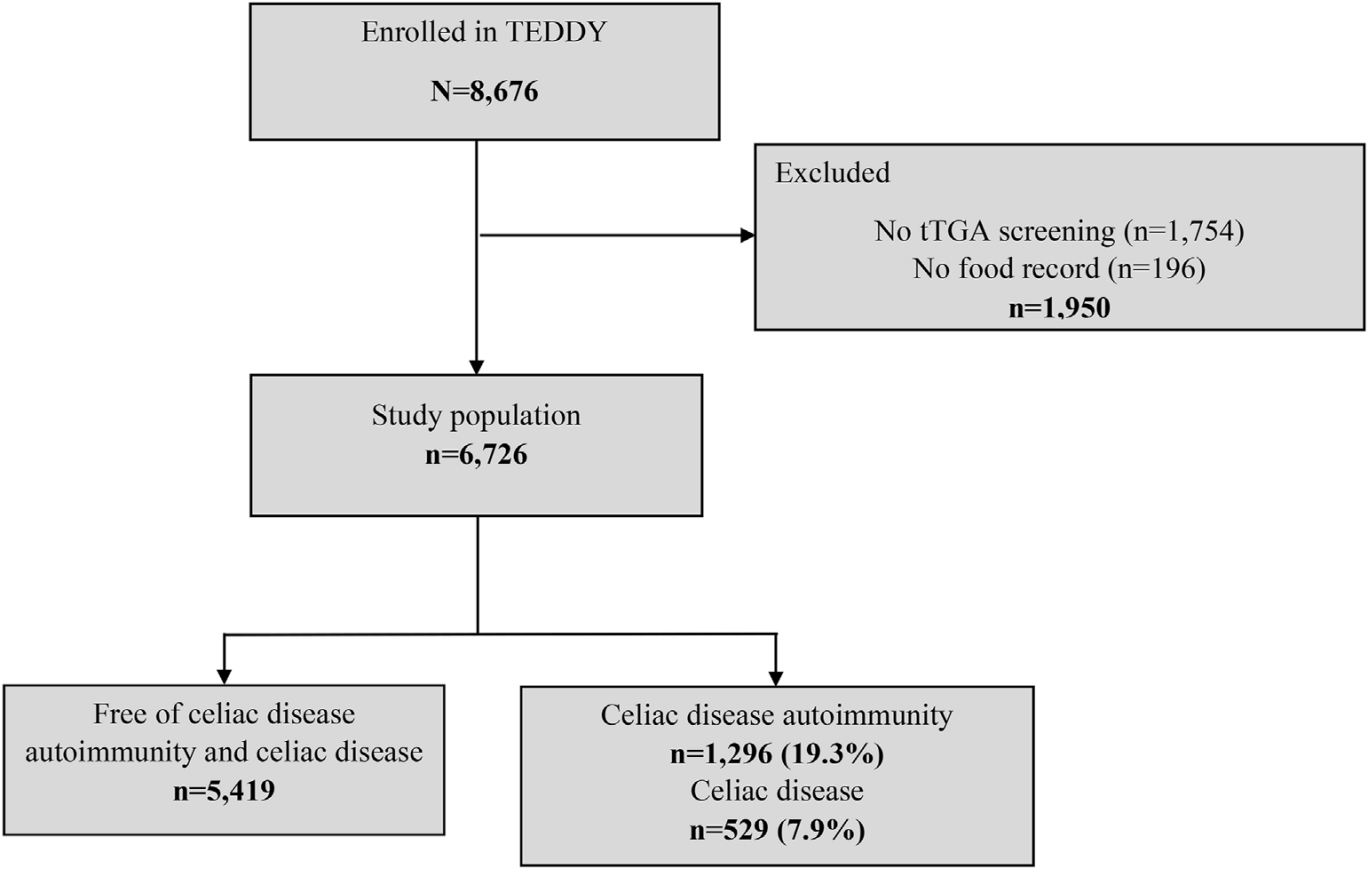
Flow chart of the study population of The Environmental Determinants of Diabetes in the Young (TEDDY) study. tTGA, tissue transglutaminase autoantibody.

TEDDY included 8676 children at genetic risk for type 1 diabetes (primary outcome) and celiac disease (secondary outcome) in a 15-y follow-up study between September 2004 and February 2010 after genetic screening at birth [16,17]. Study participants were followed at 6 clinical centers located in Colorado, Georgia, and Washington in the United States and in Sweden, Finland, and Germany in Europe. Comprehensive clinical data and research samples were prospectively collected after written informed consent from a parent or primary caregiver as previously described [16]. In all participating countries, regional or local institutional ethics board approved the study according to the Declaration of Helsinki. In the present study, 6677 children were included, with follow-up data up to 30 November, 2020, after exclusion of children without dietary data collected between age 9 to 24 mo or screening for celiac disease at follow-up (Figure 1).

### Assessment of dietary intake

Daily dietary intake was assessed by 3-d food records collected at the ages of 9, 12, 18 and 24 mo, as described in detail elsewhere [18,19]. Composite dishes were disaggregated to separate each ingredient harmonized in the TEDDY food composition database [20]. Food intake (g/d) data were aggregated into a total of 27 food groups based on nutrition profiles and culinary use (Supplemental Table 1). To account for differences in energy requirements, food group intakes were energy adjusted to g/1000 kcal/d according to the nutrient density method (food group intake/total energy intake × 1000) [21]. A total of 22,410 food records were collected for the present study. At 9-mo and 24-mo clinic visits, 92.6% and 82.8% of expected food records were completed, respectively.

### Study outcomes

Screening for celiac disease with assessment of tissue transglutaminase autoantibodies (tTGAs) started at age 24 mo and continued annually thereafter using radiobinding assays [22]. In children positive for tTGAs at age 24 mo, previously collected blood samples were analyzed to find the first time of seropositivity. CDA was defined as tTGA-positive in 2 consecutive samples at least 3 mo apart. Children with CDA were referred to their health care provider for further diagnostic evaluation of celiac disease. Celiac disease was defined as either having a small intestine biopsy showing a Marsh score ≥2, or in children who did not undergo an intestinal biopsy, having a mean tTGA concentration ≥100 U/L in 2 consecutive samples [22,23]. Using these study definitions, 1296 (19.3%) children developed CDA at a median age of 3.5 y (lower and upper quartile 2.3, 5.5) and 529 (7.9%) developed celiac disease at median age 4.7 (3.3, 6.8) y, of whom 479 (90.9%) were biopsy-confirmed (Table 1). The median time from first positive tTGA to diagnosis of celiac disease was 12.9 (7.9, 18.1) mo.

**TABLE 1:** Descriptive characteristics of included children by study outcome

	Cohort (n = 6726)	Free of CDA (n = 5419, 80.6%)	CDA (n = 1296, 19.3%)	Celiac disease (n = 529, 7.9%)
Country, n (%)				
United States	2697 (40.1)	2214 (40.9)	481 (37.1)	178 (33.6)
Sweden	2094 (31.1)	1601 (29.6)	484 (37.3)	242 (45.7)
Finland	1529 (22.7)	1261 (23.3)	265 (20.4)	89 (16.8)
Germany	406 (6.0)	339 (6.3)	66 (5.1)	20 (3.8)
Female, n (%)	3291 (48.9)	2549 (47.0)	735 (56.7)	319 (60.3)
HLA genotype, n (%)				
DQ2/DQ2^1^	1397 (20.8)	864 (15.9)	526 (40.6)	262 (49.5)
DQ2/DQ8^2^	2637 (39.2)	2130 (39.3)	504 (38.9)	180 (34.0)
Other HLA antigen genotypes^3^	2692 (40.0)	2425 (44.7)	266 (20.5)	87 (16.4)
First-degree relative with celiac disease, n (%)	329 (4.9)	173 (3.2)	155 (12.0)	94 (17.8)

CDA, celiac disease autoimmunity; HLA, human leucocyte antigen.

Percentages may not add to 100% due to rounding.

1 Including genotype DR3∗0501/0201∗DR3∗0501/0201

2 Including genotype DR4∗030X/0302∗DR3∗0501/0201

3 Including genotypes DR4∗030X/0302∗DR4∗030X/0302, DR4∗030X/0302∗DR4∗030X/020X, DR4∗030X/0302∗DR8∗0401/0402, DR4∗030X/0302∗DR1∗0101/0501, DR4∗030X/0302∗DR13∗0102/0604, DR4∗030X/0302∗DR4∗030X/0304, DR4∗030X/0302∗DR9∗030X/0303, DR3∗0501/0201∗DR9∗030X/0303.

### Statistical analysis

#### Dietary pattern analysis

PCA [24] was performed to derive dietary patterns present in the study population at age 9, 12, 18, and 24 mo. Briefly, this method reduced a large set of predictors (food groups) to a smaller number of components (dietary patterns) based on linear combinations of the predictors. This explorative method derived dietary patterns explaining as much variation in the food groups as possible [25]. Food groups with less than 25% consumers at each visit were excluded, to minimize the effect of zero inflated distributions. Children with CDA, celiac disease, or type 1 diabetes at the time of the dietary assessment were not included in the PCA analyses. Components were extracted based on established criteria; the Kaiser Meier Olkin test for sample adequacy, scree plot examination, and component eigenvalue of >1.0 [[26], [27], [28]]. Varimax rotation was applied to create independent dietary patterns. Factor loadings representing correlations between the food groups with each component were computed and food groups without a loading of ≥0.2/≤−0.2 in any of the extracted components were removed. Dietary patterns were named based on the 2 food groups with the highest positive factor loadings in the component. To increase interpretability and comparability, the method of constructing so-called simplified dietary patterns was applied [29] in which only food groups with an absolute factor loading of ≥0.2/≤−0.2 were retained. One adherence score per dietary pattern was calculated for each child that reflected the similarity of their dietary intake with the respective dietary pattern.

#### Association analyses

Cox proportional hazards regression [30] was used to examine associations between adherence to dietary patterns at each age separately and risk of CDA as well as celiac disease, respectively. Dietary pattern adherence scores were modeled as time dependent, continuous variables. Time to CDA was defined as age at the first of the consecutive positive tTGA samples. The right-censoring was age at the last negative tTGA sample measured. Time to celiac disease was age at diagnosis and right-censoring age at the last study visit prior to diagnosis. Models were adjusted for previously reported risk factors associated with celiac disease [22]: HLA genotype, female sex, having a parent or sibling with celiac disease, and country of residence (United States as reference). Total daily energy intake (kcal/d) assessed at the corresponding food record was included to adjust for possible confounding by energy intake [21]. Only cases with complete data were included in the association analyses. To estimate if the association of adherence to dietary patterns was independent of amount of gluten intake, models were further adjusted for total gluten intake (g/d) [2]. Schoenfeld residuals for each model were examined to evaluate the model fit. Effect sizes from the Cox models were described as hazard ratios (HRs) with their related 95% confidence intervals (CIs) and expressed per 5 unit increase in adherence to the dietary pattern. All tests were 2-sided, and *P* values of <0.05 were considered statistically significant. SPSS (IBM SPSS, version 27.0, IMB Corp) and SAS 9.4 (SAS/STAT, version 15.2, SAS Institute Inc) were used for all analyses.

## Results

### Overall dietary patterns

At each age of 9, 12, and 24 mo, 3 dietary patterns in the study population were identified by PCA (Supplemental Table 2). The total variance in food intake explained by the identified dietary patterns was 35.8% at age 9 mo, 31.3% at age 12 mo, and 32.2% at age 24 mo. At age 18 mo, 4 dietary patterns were identified by PCA with a total variance of 24.9%. Dietary patterns with higher intake of the food groups Vegetable fats and Wheat and low intakes of Legumes were found at all ages, as were patterns with higher intake of the food groups Fruit, Vegetables, Legumes, and Root vegetables and patterns with higher intake of the food groups Potatoes, Meat, Root vegetables, Rice and gluten-free (GF) grains and lower intake of Cheese (Table 2). The mean adherence scores to the dietary patterns varied by country (Supplemental Table 3). Dietary patterns with higher intakes of the food groups Potatoes, Meat, Rye and barley, Root Vegetables, Rice and GF grains and lower intakes of Cheese, were most common in Finnish children and were uncommon in US children. Dietary patterns with higher intakes of the food groups Vegetable fats, the gluten-containing grains Wheat and Rye and barley, and Juice and lower intakes of Legumes were most common in Swedish children and uncommon in US children.

**TABLE 2:** Characteristics of dietary patterns simplified from principal components analyses on food intake assessed by 3-d food records

Age, mo	Dietary pattern	Higher intake of food group	Lower intake of food group
9	“Vegetable fats and Milk”	Vegetable fats, Milk, Wheat, Rice and GF grains, Oats, Juices, Potatoes	Infant formula, Human milk, Legumes
	“Potatoes and Meat”	Potatoes, meat, Rye and barley, Root vegetables, Rice and GF grains, Sugar and sweets, Vegetables, Oats	Cheese
	“Fruit and Vegetables”	Fruit and berries, Vegetables, Legumes, Root vegetables, Animal fats, Cheese, Fermented dairy, Wheat	
12	“Vegetable fats and Wheat”	Vegetable fats, Wheat, Rye and barley, Milk, Processed meat, Fish and seafood, Juices, Rice and GF grains	Root vegetables, Legumes, Infant formula
	“Potatoes and Oats”	Potatoes, Oats, Rice and GF grains, Meat, Root vegetables, Rye and barley, Fish and seafood	Cheese, Eggs, Processed meat, Wheat, Legumes
	“Vegetables and Fruit”	Vegetables, Fruit and berries, Root vegetables, Rice and GF grains, Juices, Wheat	Milk, Fermented dairy
18	“Wheat and Vegetable fats”	Vegetable fats, Wheat, Processed meats, Juices, Rye and barley	Milk, Oats, Meat, Legumes
	“Meat, Rice and GF grains”	Meat, Rice and GF grains, Potatoes, Root vegetables, Sweet beverages	Milk, Cheese, Wheat
	“Fruit and Vegetables”	Fruit and berries, Vegetables, Root vegetables, Legumes, Fish and seafood	Sweet beverages, Sugar and sweets
	“Rye, barley, and Vegetable fats”	Rye and barley, Vegetable fats, Potatoes, Milk, Oats, Fish and seafood, Fermented dairy	Sugar and sweets, Nuts and seeds, Legumes, Cheese, Eggs, Sweet beverages, Animal fats
24	“Wheat and Vegetable fats”	Wheat, Vegetable fats, Juices, Processed meats, Rye and barley	Milk, Meat, Oats, Legumes, Root vegetables
	“Rye, barley, and Potatoes”	Rye and barley, Potatoes, Vegetable fats, Oats, Root vegetables, Fish and seafood, Fermented dairy, Meat, Rice and GF grains, Vegetables	Sugar and sweets, Lite beverages, Nuts and seeds, Cheese, Eggs, Legumes
	“Fruit and Vegetables”	Fruit and berries, Vegetables, Legumes, Cheese, Root vegetables, Nuts and seeds	Milk, Sweet beverages, Potatoes, Ice cream

GF, gluten-free.

### Associations between dietary patterns and CDA

Adherence to the dietary pattern *“Vegetable fats and Milk”* at age 9 mo was associated with the reduced risk of CDA during follow-up (HR: 0.90; 95% CI: 0.81, 0.99; *P* = 0.04, per 5-unit increased adherence), and the association remained after adjusting for total daily gluten intake (HR: 0.88; 95% CI: 0.79, 0.98; *P* = 0.02, per 5-unit increased adherence) (Table 3). Interaction analysis showed that the association was found in children from the United States and Finland (HR: 0.67; 95% CI: 0.54, 0.83 and HR: 0.76; 95% CI: 0.60, 0.87; respectively, *P* = 0.009 for interaction), and in those carrying HLA DQ2/DQ8 (HR: 0.77; 95% CI: 0.68, 0.88; *P* = 0.001 for interaction) (Supplemental Table 4).

**TABLE 3:** Cox proportional hazards estimated HRs with related 95% CIs of the association between dietary patterns derived by principal comonents analyses and risk of celiac disease autoimmunity in 6677 children at genetic risk

Dietary pattern	Association with celiac disease autoimmunity *Per 5-unit increased adherence score*				
	Events	HR (95% CI)	*P*	Adjusted for gluten intake HR (95% CI)	*P*
Age 9 mo	*n* = 1216				
“Vegetable fats and Milk”		0.90 (0.81, 0.99)	0.04	0.88 (0.79, 0.98)	0.02
“Potatoes and Meat”		1.02 (0.92, 1.14)	0.71	1.03 (0.92, 1.15)	0.60
“Fruit and Vegetables”		1.04 (0.96, 1.14)	0.33	1.02 (0.93, 1.12)	0.65
Age 12 mo	*n* = 1177				
“Vegetable fats and Wheat”		0.98 (0.90, 1.07)	0.68	0.96 (0.87, 1.05)	0.34
“Potatoes and Oats”		0.94 (0.87, 1.03)	0.18	0.97 (0.88, 1.06)	0.44
“Vegetables and Fruit”		0.98 (0.89, 1.08)	0.72	0.96 (0.87, 1.07)	0.45
Age 18 mo	*n* = 1085				
“Wheat and Vegetable fats”		1.13 (1.01, 1.26)	0.04	1.03 (0.91, 1.17)	0.63
“Meat, Rice and GF grains”		0.91 (0.82, 1.01)	0.67	0.96 (0.86, 1.07)	0.48
“Fruit and Vegetables”		1.05 (0.95, 1.16)	0.32	1.03 (0.93, 1.14)	0.55
“Rye, barley and Vegetable fats”		1.00 (0.92, 1.09)	0.999	1.01 (0.92, 1.10)	0.91
Age 24 mo	*n* = 903				
“Wheat and Vegetable fats”		1.29 (1.16, 1.44)	<0.001	1.18 (1.05, 1.33)	<0.001
“Rye, barley and Potatoes”		1.07 (0.99, 1.16)	0.09	1.07 (0.98, 1.16)	0.13
“Fruit and Vegetables”		1.05 (0.96, 1.15)	0.32	1.02 (0.92, 1.12)	0.75

CI, confidence interval; GF, gluten-free; HR, hazard ratio.

At the ages of 18 and 24 mo, adherence to the dietary patterns *“Wheat and vegetable fats”* were associated with the increased risk of CDA (HR: 1.13; 95% CI: 1.01, 1.26; *P* = 0.04 and HR: 1.29; 95% CI: 1.16, 1.44; *P* < 0.001, respectively, per 5-unit increased adherence). However, the association only remained at age 24 mo after adjusting for the total daily gluten intake (HR: 1.18; 95% CI: 1.05, 1.33; *P* < 0.001, per 5-unit increased adherence). Interaction analysis showed that the association at 24 mo was found in children with genotypes other than DQ2/DQ2 and DQ2/DQ8 (HR: 1.41; 95% CI: 1.17, 1.70; *P* = 0.04 for interaction).

### Associations between dietary patterns and celiac disease

Adherence at age 18 mo to the dietary pattern *“Meat, Rice and GF grains”* was inversely associated with risk of celiac disease (HR: 0.79; 95% CI: 0.67, 0.93; *P* = 0.01, per 5-unit increased adherence), and the association remained after adjusting for total daily gluten intake (HR: 0.83; 95% CI: 0.70, 0.99; *P* = 0.04, per 5-unit increased adherence) (Table 4). The association was observed in children with HLA DQ2/DQ2 (HR: 0.75; 95% CI: 0.60, 0.94) and HLA other than DQ2/DQ8 (HR: 0.68; 95% CI: 0.47, 0.995; *P* = 0.04 for interaction) (Supplemental Table 4).

**TABLE 4:** Cox proportional hazards estimated HRs with related 95% CIs of the association between dietary patterns derived by principal components analyses and risk of celiac disease in 6677 children at genetic risk

Dietary pattern	Association with celiac disease *Per 5-unit increased adherence score*				
	Events	HR (95% CI)	*P*	Adjusted for gluten intake HR (95% CI)	*P*
Age 9 mo	*n* = 501				
“Vegetable fats and Milk”		0.92 (0.79, 1.08)	0.30	0.90 (0.77, 1.06)	0.22
“Potatoes and Meat”		1.03 (0.87, 1.23)	0.72	1.04 (0.88, 1.24)	0.64
“Fruit and Vegetables”		1.05 (0.92, 1.20)	0.46	1.03 (0.89, 1.19)	0.71
Age 12 mo	*n* = 494				
“Vegetable fats and Wheat”		1.05 (0.92, 1.20)	0.45	1.03 (0.90, 1.19)	0.67
“Potatoes and Oats”		0.94 (0.82, 1.07)	0.32	0.95 (0.83, 1.09)	0.48
“Vegetables and Fruit”		0.94 (0.81, 1.09)	0.39	0.92 (0.79, 1.08)	0.31
Age 18 mo	*n* = 440				
“Wheat and Vegetable fats”		1.02 (0.86, 1.24)	0.83	0.95 (0.78, 1.16)	0.62
“Meat, Rice and GF grains”		0.79 (0.67, 0.93)	0.01	0.83 (0.70, 0.99)	0.04
“Fruit and Vegetables”		0.95 (0.81, 1.11)	0.51	0.93 (0.79, 1.10)	0.39
“Rye, barley and Vegetable fats”		1.08 (0.94, 1.24)	0.26	1.09 (0.95, 1.25)	0.24
Age 24 mo	*n* = 362				
“Wheat and Vegetable fats”		1.38 (1.16, 1.64)	<0.001	1.24 (1.03, 1.50)	0.03
“Rye, barley and Potatoes”		1.07 (0.94, 1.22)	0.32	1.06 (0.93, 1.21)	0.38
“Fruit and Vegetables”		0.94 (0.81, 1.10)	0.43	0.90 (0.78, 1.06)	0.20

CI, confidence interval; GF, gluten-free; HR, hazard ratio.

The dietary pattern *“Wheat and Vegetable fats”* at age 24 mo was associated with an increased risk of celiac disease (HR: 1.38; 95% CI: 1.16, 1.64; *P* < 0.001, per 5-unit increased adherence). This association remained after adjustment for total daily gluten intake (HR: 1.24; 95% CI: 1.03, 1.50; *P* = 0.03, per 5-unit increased adherence). For this dietary pattern, there were no interactions with other included factors found with risk of celiac disease.

## Discussion

The main finding of this study was that dietary patterns identified in the study population during the 2 first years in life were associated with the subsequent risk of CDA and celiac disease in children at genetic risk. The associations observed were independent of the amount of gluten intake, indicating that additional dietary factors after weaning may have implications on the incidence of CDA and celiac disease in childhood. Moreover, differences in adherence to the observed dietary patterns by country may partly explain the regional variations in incidence of CDA and celiac disease in TEDDY [7].

The dietary pattern *“Wheat and Vegetable fats”* at age 24 mo was associated with increased risk of both study outcomes, and the gluten intake from this pattern further attenuated the association. This was in line with an Italian study in which a more Western-like diet with higher intakes of wheat and juice and lower intakes of legumes and milk in the second year of life were demonstrated in children later diagnosed with celiac disease [4]. A Western diet and lifestyle have been hypothesized to be involved in the pathophysiology of celiac disease via adverse effects on the intestinal microbiota and immune system [31], and together with the observations of this study, it thus warrants further investigation. Conversely, our findings overlap with a previous study in which a “prudent” dietary pattern after weaning, higher in potatoes, oats, rice, and meat, demonstrated a lower risk of CDA [15]. However, in the present study, health-conscious dietary patterns with higher intakes of fruits, vegetables, fish and seafood, and legumes, were not associated with the study outcomes. Contrastingly, higher maternal fiber intake from fruits (which may serve as a proxy to offspring intake) was associated with a reduced risk of celiac disease in the offspring in a Norwegian population-based cohort [32].

Using a data-driven approach, specific dietary patterns in children at genetic risk thus seem to associate with risk of CDA and celiac disease independent of the amount of gluten in their diet. The nutritional intake from the dietary patterns was not within the scope of the present study, but investigating intakes of various micronutrients, fatty acids, carbohydrates, and dietary fiber may further add to the understanding of these associations.

There were similarities in the dietary patterns found across the ages; however, with increasing age, more food groups were included in the dietary patterns. This observed variation reflects the transition process after weaning to established dietary habits at around age 2 y [33,34]. The dietary pattern at age 9 mo associated with the lower risk of CDA was characterized by lower intakes of both Human milk as well as Infant formula. However, neither human milk nor formula type has previously been associated with celiac disease [[35], [36], [37]]. Some food groups were included in dietary patterns with conflicting associations with the study outcomes. These contradicting findings may be explained by differences in the foods eaten from each food group depending on the child’s age. For instance, nutrient-dense infant cereals were in Swedish children in TEDDY a common source of both wheat, and dairy at age 9, while other, less nutrient-dense wheat-based foods are more commonly consumed at older ages [38].

This study has several limitations. PCA empirically investigates dietary patterns present in a study population and is suggested for an explorative approach [9,39]. The dietary patterns found by PCA represented about one-third of the variance in dietary intake, which is high compared with similar studies including children. However, the patterns identified in the present study may be specific for the TEDDY cohort and not comparable with that of the general population [40]. Moreover, they may not capture the dietary patterns or specific dietary factor most likely to be associated with the study outcomes [41]. However, methods for deriving dietary patterns that best predict disease outcome may not be representative in the study population [41,42]. Since PCA is designed for cross-sectional data and the fact that there was a variation in the dietary patterns found across the observed ages, this restricted the present study to investigate the different dietary patterns longitudinally.

Using the method of simplified dietary patterns may instead be considered a strength of this data-driven study, as these are less population-dependent compared to other methods and thus allow for validation and reproduction in other populations [29]. Investigating dietary patterns as well as simplified patterns, however, does not capture variation in intake of single foods or foods not included in the pattern studied. The comprehensive data collection in the TEDDY cohort with repeated exposure that captures variation over time and outcome assessment and highly detailed data [16] allowed for examining dietary patterns at specific ages while including information on relevant confounders and risk factors with statistical power.

In conclusion, dietary patterns higher in the food groups Wheat, Vegetable fats, Juice, and Processed meats and lower in Milk, Meat, Oats, and Legumes in the second year of life were associated with an increased risk of CDA and celiac disease. Although gluten intake is critical in affecting risk of celiac disease in early childhood, nongluten dietary factors should also be considered, and more research is needed to further define these associations in children at genetic risk.

### Author contributions

The authors’ responsibilities were as follows—EMH, LKM: had full access to all the data and take full responsibility for the integrity of the data and the accuracy of the data analysis; EMH, LKM, XL, UU, JY, JN, SMV, EL, KK, CA, DA: designed the research; all authors: conducted the research; EMH, LKM: analyzed the data; EMH, LKM, DA: drafted the paper; EMH, LKM, DA: had primary responsibility for the final content; and all authors: read and approved the final manuscript.

### Conflicts of interest

The authors report no conflicts of interest.

### Funding

This study was funded by the Swedish Research Council (Grant 2018-02553). The TEDDY studyis funded by U01 DK63829, U01 DK63861, U01 DK63821, U01 DK63865, U01 DK63863, U01 DK63836, U01 DK63790, UC4 DK63829, UC4 DK63861, UC4 DK63821, UC4 DK63865, UC4 DK63863, UC4 DK63836, UC4 DK95300, UC4 DK100238, UC4 DK106955, UC4 DK112243, UC4 DK117483, U01 DK124166, U01 DK128847, and Contract No. HHSN267200700014C from the National Institute of Diabetes and Digestive and Kidney Diseases (NIDDK), National Institute of Allergy and Infectious Diseases (NIAID), Eunice Kennedy Shriver National Institute of Child Health and Human Development (NICHD), National Institute of Environmental Health Sciences (NIEHS), Centers for Disease Control and Prevention (CDC), and JDRF. This work is supported in part by the NIH/NCATS Clinical and Translational Science Awards to the University of Florida (UL1 TR000064) and the University of Colorado (UL1 TR002535). The content is solely the responsibility of the authors and does not necessarily represent the official views of the National Institutes of Health.

### Data availability

The datasets generated and analyzed during the current study will be made available in the NIDDK Central Repository at https://repository.niddk.nih.gov/studies/teddy.

## Appendix A Supplementary data

Multimedia component1 Multimedia component1

Multimedia component2 Multimedia component2

Multimedia component3 Multimedia component3

Multimedia component4 Multimedia component4

Multimedia component5 Multimedia component5
