# Supplementary material for: Associations of dietary patterns between age 9 and 24 months with risk of celiac disease autoimmunity and celiac disease among children at increased risk
Source: Am J Clin Nutr. 2023 Oct 16;118(6):1099–105. doi: 10.1016/j.ajcnut.2023.08.009 (PMC10925856; doi:10.1016/j.ajcnut.2023.08.009)
Supplement: Multimedia component1 [file mmc1.docx]

**Online Supplemental material**

**Title**
Associations of adherence to dietary patterns between age 9 and 24 months with the risk of celiac disease autoimmunity and celiac disease among children at increased risk.

**Authors**
EM Hård af Segerstad et al.

**eTable 1 Aggregated food groups used in dietary pattern analysis.**

**eTable 1 Aggregated food groups used in dietary patterns analysis.**

| Food group | Included foods |
| --- | --- |
| Wheat | Various types of wheat (e.g., flour, flakes) |
| Rye and barley | Various types of rye and barley (e.g., flour, flakes) |
| Oats | Dry oats, oat milk (converted to dry oats) |
| Rice and  gluten-free grains | Rice (cooked, flour, milk, converted to dry weight), and gluten-free grains, flour, and starches (e.g., corn (flakes, meal, polenta, popcorn), millet, buckwheat, quinoa, potato flour starch, wheat starch) |
| Potatoes | Potatoes cooked using different methods (e.g., cooked, fried, chips, baked) |
| Root vegetables | All root vegetables (e.g., sweet potato, carrot, turnip, rutabaga). |
| Vegetables | All vegetables (e.g., leafy vegetables, onions, cabbages, mushrooms, fruit vegetables), fresh, canned, and dried. |
| Fruits and berries | All fruits and berries (e.g., apple, banana, pear, citrus, strawberries), fresh, canned, and dried. |
| Juices | All fruit, berry, and vegetable juices. |
| Nuts and seeds | All nuts and seeds, included raw and roasted, spreads. Nut and seed milks converted to solid nuts and seeds. |
| Legumes | All legumes (e.g., beans, peas, soybeans), fresh, canned. Dried converted to fresh. Soy milk converted to raw soybeans. |
| Meat | Meat and organ meats of various sources (e.g., pork, beef, lamb, poultry). |
| Processed meat | Meat and sausages of various type and source (e.g., sausages, cold-cuts, bacon, canned meat). |
| Fish and seafood | All types from various sources, fresh, frozen, processed, and canned. |
| Eggs | All types from various poultry. Powders are converted to raw. |
| Milk | All types of animal milk, cream with different fat content. Includes whey, milk powders. Powders are converted to liquids. |
| Fermented dairy | Fermented milk products (e.g., sour milk, yoghurt, sour cream, crème fraiche). Powders are converted to liquids. |
| Cheese | Fresh and aged cheese |
| Ice cream | Dairy ice creams |
| Non-dairy | Yoghurts, ice cream, kefir etc. from non-dairy origin such as soy, rice, oats, and coconuts. |
| Human milk | Human breastmilk |
| Infant formula | All types of infant formula (from cow, other animal, or soy, partially and fully hydrolyzed). Powders converted to liquid. |
| Vegetable fats | Fats of various vegetable origin (e.g., canola, olive, corn, nuts), margarine and margarine-butter spreads with various fat content. |
| Animal fats | Butter and animal fats (lard) |
| Sweet beverages | Sugar sweetened beverages (e.g., soft drinks, fruit and berry drinks, nectar). |
| Lite beverages | Unsweetened, artificially sweetened, and low-calorie sweetened (e.g., Stevia) drinks (e.g., soft drink, fruit and berry drinks, nectar), coffee and tea. |
| Sugar and sweets | Including sugar candy, energy bars, chocolate, sugar, syrup, honey, jams, and jellies. |
