# Supplementary material for: Associations of dietary patterns between age 9 and 24 months with risk of celiac disease autoimmunity and celiac disease among children at increased risk
Source: Am J Clin Nutr. 2023 Oct 16;118(6):1099–105. doi: 10.1016/j.ajcnut.2023.08.009 (PMC10925856; doi:10.1016/j.ajcnut.2023.08.009)
Supplement: Multimedia component2 [file mmc2.docx]

**Online Supplemental material**

**Title**Associations of adherence to dietary patterns between age 9 to 24 months with the risk of celiac disease autoimmunity and celiac disease among children at increased risk.

**Authors**EM Hård af Segerstad et al.

**eTable 2 Factor loadings of food groups in dietary patterns derived by principal component analysis on dietary intake assessed by 3-day food records at age 9 to 24 months in children at genetic risk of type 1 diabetes and celiac disease.**

|  | Dietary patterns | | | | | | | | | | | | |
| --- | --- | --- | --- | --- | --- | --- | --- | --- | --- | --- | --- | --- | --- |
|  | *Veg. fats, milk* | *Potatoes, meat* | *Fruit, veg.* | *Veg. fats, wheat* | *Potatoes, oats* | *Veg., fruit* | *Wheat, veg. fats* | *Meat, rice* | *Fruits, veg.* | *Rye, veg. fats* | *Wheat, veg. fats* | *Rye, potatoes* | *Fruits, veg.* |
| Age, months | 9 (n=6146) | | | 12 (n=5866) | | | 18 (n=5315) | | | | 24 (n=4876) | | |
| Food group |  |  |  |  |  |  |  |  |  |  |  |  |  |
| Wheat | 0.702 | - | 0.233 | 0.703 | -0.273 | 0.236 | 0.704 | -0.204 | - | - | 0.687 | - | - |
| Rye, barley | - | 0.549 | - | 0.362 | 0.399 | - | 0.253 | - | - | 0.587 | 0.229 | 0.623 | - |
| Oats | 0.469 | 0.204 | - | - | 0.552 | - | -0.255 | - | - | 0.402 | -0.286 | 0.407 | - |
| Rice, GF grain | 0.545 | 0.326 | - | 0.213 | 0.438 | 0.374 | - | 0.522 | - | - | - | 0.269 | - |
| Potatoes | 0.213 | 0.712 | - | - | 0.633 | - | - | 0.466 | - | 0.442 | - | 0.554 | -0.222 |
| Root vegetables | - | 0.349 | 0.438 | -0.206 | 0.397 | 0.405 | - | 0.385 | 0.353 | - | -0.255 | 0.376 | 0.257 |
| Vegetables | - | 0.208 | 0.511 | - | - | 0.591 | - | - | 0.568 | - | - | 0.222 | 0.550 |
| Fruits, berries | - | - | 0.676 | - | - | 0.485 | - | - | 0.640 | - | - | - | 0.603 |
| Juices | 0.410 | - | - | 0.262 | - | 0.318 | 0.380 | - | - | - | 0.395 | - | - |
| Nuts and seeds | * | * | * | * | * | * | - | - | - | -0.432 | - | -0.384 | 0.226 |
| Legumes | -0.369 | - | 0.452 | -0.356 | -0.230 | - | -0.232 | - | 0.274 | -0.386 | -0.270 | -0.215 | 0.382 |
| Meat | - | 0.678 | - | - | 0.450 | - | -0.230 | 0.576 | - | - | -0.367 | 0.295 | - |
| Processed meat | * | * | * | 0.322 | -0.285 | - | 0.387 | - | - | - | 0.355 | - | - |
| Fish, seafood | * | * | * | 0.264 | 0.255 | - | - | - | 0.229 | 0.293 | - | 0.349 | - |
| Eggs | * | * | * | - | -0.366 | - | - | - | - | -0.287 | - | -0.249 | - |
| Milk | 0.856 | - | - | 0.380 | - | -0.491 | -0.552 | -0.464 | - | 0.428 | -0.427 | - | -0.384 |
| Fermented dairy | - | - | 0.311 | - | - | -0.300 | - | - | - | 0.266 | - | 0.319 | - |
| Cheese | - | -0.201 | 0.351 | - | -0.473 | - | - | -0.290 | - | -0.347 | - | -0.368 | 0.279 |
| Ice cream | * | * | * | * | * | * | * | * | * | * | - | - | -0.214 |
| Non-dairy | * | * | * | * | * | * | * | * | * | * | * | * | * |
| Human milk | -0.387 | - | - | * | * | * | * | * | * | * | * | * | * |
| Infant formula | -0.517 | - | - | -0.659 | - | - | * | * | * | * | * | * | * |
| Vegetable fats | 0.893 | - | - | 0.768 | - | - | 0.559 | - | - | 0.486 | 0.478 | 0.452 | - |
| Animal fats | - | - | 0.366 | - | - | - | - | - | - | -0.223 | - | - | - |
| Sweet beverages | * | * | * | - | - | - | - | 0.310 | -0.505 | -0.234 | - | - | -0.362 |
| Light beverages | * | * | * | * | * | * | * | * | * | * | - | -0.409 | - |
| Sugar, sweets | - | 0.238 | - | - | - | - | - | - | -0.276 | -0.546 | - | -0.523 | - |
| Variance explained | 17.8% | 9.6% | 8.4% | 12.2% | 11.3% | 7.8% | 7.9% | 7.0% | 6.7% | 10.6% | 7.3% | 11.0% | 6.7% |

*= <25% consumers at the visit, food group was excluded from analysis.
- =Factor loading <2.0 to >-0.2.
*Abbreviations: GF=gluten-free, veg=vegetable*
