# Supplementary material for: Associations of dietary patterns between age 9 and 24 months with risk of celiac disease autoimmunity and celiac disease among children at increased risk
Source: Am J Clin Nutr. 2023 Oct 16;118(6):1099–105. doi: 10.1016/j.ajcnut.2023.08.009 (PMC10925856; doi:10.1016/j.ajcnut.2023.08.009)
Supplement: Multimedia component3 [file mmc3.docx]

**Online Supplemental material**

**Title**
Associations of adherence to dietary patterns between age 9 to 24 months with the risk of celiac disease autoimmunity and celiac disease among children at increased risk.

**Authors**
EM Hård af Segerstad et al.

**eTable 3** **Mean adherence score by country to dietary patterns extracted by principal components analysis in children at genetic risk of type 1 diabetes and celiac disease. A higher score reflects closer similarity with the dietary pattern.**

|  | Mean adherence score (SD) | | | |
| --- | --- | --- | --- | --- |
| Dietary pattern | US | Sweden | Finland | Germany |
| Age 9 months | n=2389 | n=1987 | n=1427 | n=343 |
| *“Vegetable fats and Milk”* | -5.3 (2.3) | 5.7 (3.9) | 0.6 (2.8) | 1.5 (3.9) |
| *“Potatoes and Meat”* | -2.6 (3.1) | 0.5 (2.8) | 3.4 (3.6) | 1.0 (3.4) |
| *“Fruit and Vegetables”* | 0.3 (3.8) | 0.5 (3.5) | -1.5 (1.9) | 1.6 (4.2) |
|  |  |  |  |  |
| Age 12 months | n=2271 | n=1925 | n=1359 | n=311 |
| *“Vegetable fats and Wheat”* | -3.7 (3.6) | 4.2 (3.1) | 0.1 (3.3) | 0.7 (4.0) |
| *“Potatoes and Oats”* | -3.7 (3.4) | 0.7 (3.4) | 5.2 (3.5) | -0.3 (4.0) |
| *“Vegetables and Fruit”* | -1.2 (2.9) | 1.4 (3.1) | -0.7 (3.1) | 3.7 (3.6) |
|  |  |  |  |  |
| Age 18 months | n=2076 | n=1746 | n=1240 | n=253 |
| *“Wheat and Vegetable fats”* | -2.2 (2.7) | 3.1 (2.8) | -1.5 (3.2) | 3.8 (3.3) |
| *“Meat, Rice and GF grains”* | -1.4 (3.2) | 0.2 (2.8) | 2.3 (3.3) | -0.8 (3.5) |
| *“Fruit and Vegetables”* | -0.5 (3.4) | 0.5 (2.9) | 0.2 (2.8) | -0.5 (3.8) |
| *“Rye, barley and Vegetable fats”* | -4.8 (3.8) | 3.2 (3.2) | 4.2 (3.8) | -4.4 (5.0) |
|  |  |  |  |  |
| Age 24 months | n=1952 | n=1609 | n=1103 | n=212 |
| *“Wheat and Vegetable fats”* | -2.6 (3.0) | 2.8 (3.2) | -1.3 (3.6) | 3.5 (3.3) |
| *“Rye, barley and Potatoes”* | -5.5 (4.0) | 3.4 (3.7) | 5.0 (4.2) | -2.5 (4.3) |
| *“Fruit and Vegetables”* | 1.7 (4.1) | -1.0 (3.0) | -1.8 (3.3) | 1.6 (4.1) |

*Abbreviation: GF, gluten-free*
