# Supplementary material for: Associations of dietary patterns between age 9 and 24 months with risk of celiac disease autoimmunity and celiac disease among children at increased risk
Source: Am J Clin Nutr. 2023 Oct 16;118(6):1099–105. doi: 10.1016/j.ajcnut.2023.08.009 (PMC10925856; doi:10.1016/j.ajcnut.2023.08.009)
Supplement: Multimedia component4 [file mmc4.docx]

**Online Supplemental material**

**Title**Associations of adherence to dietary patterns between age 9 to 24 months with the risk of celiac disease autoimmunity and celiac disease among children at increased risk.

**Authors**
EM Hård af Segerstad et al.

**eTable 4 Estimates of interaction analyses between adherence to dietary patterns, covariates and associations with celiac disease autoimmunity and celiac disease in children at genetic risk.**

**eTable 3 Estimates of interaction analyses between dietary patterns, covariates and associations with celiac disease autoimmunity and celiac disease in children at genetic risk.**

|  | | | Celiac disease autoimmunity | | Celiac disease | |
| --- | --- | --- | --- | --- | --- | --- |
|  |  |  | Wald test | Effect of 5-units increased adherence | Wald test | Effect of 5-units increased adherence |
| Interaction Terms | Age, months | Category | *P interaction* | aHR (95%CI)^a^ | *P interaction* | aHR (95%CI)^a^ |
| Country* *Vegetable fats and Milk* | 9 | USA | .009 | 0.67 (0.54, 0.83) | .20 |  |
|  |  | Sweden |  | 0.99 (0.87, 1.13) |  |  |
|  |  | Finland |  | 0.76 (0.60, 0.87) |  |  |
|  |  | Germany |  | 0.92 (0.65, 1.30) |  |  |
| HLA genotype* *Vegetable fats and Milk* | 9 | DQ2/DQ2^b^ | .001 | 0.93 (0.82, 1.05) | .004 | 0.90 (0.75, 1.08) |
|  |  | DQ2/DQ8^c^ |  | 0.77 (0.68, 0.88) |  | 0.77 (0.63, 0.94) |
|  |  | Other^d^ |  | 0.96 (0.83, 1.11) |  | 1.14 (0.91, 1.42) |
| HLA genotype* *Vegetable fats and Wheat* | 12 | DQ2/DQ2^b^ | .01 | 1.02 (0.91, 1.13) | .04 | 1.08 (0.92, 1.27) |
|  |  | DQ2/DQ8^c^ |  | 0.85 (0.75, 0.96) |  | 0.88 (0.72, 1.07) |
|  |  | Other^d^ |  | 1.02 (0.87, 1.19) |  | 1.23 (0.94, 1.60) |
| HLA genotype*  *Vegetables and Fruit* | 12 | DQ2/DQ2^b^ | .01 | 1.10 (0.95, 1.27) | .46 |  |
|  |  | DQ2/DQ8^c^ |  | 0.80 (0.69, 0.93) |  |  |
|  |  | Other^d^ |  | 1.03 (0.85, 1.24) |  |  |
| HLA genotype* *Wheat and Vegetable fats* | 18 | DQ2/DQ2^b^ | .02 | 1.07 (0.91, 1.26) | .02 | 0.99 (0.77, 1.26) |
|  |  | DQ2/DQ8^c^ |  | 0.91 (0.78, 1.07) |  | 0.78 (0.60, 1.01) |
|  |  | Others^d^ |  | 1.21 (1.00, 1.47) |  | 1.28 (0.92, 1.77) |
| HLA genotype* *Meat, Rice and GF grains* | 18 | DQ2/DQ2^b^ | .82 |  | .04 | 0.75 (0.60, 0.94) |
|  |  | DQ2/DQ8^c^ |  |  |  | 1.09 (0.83, 1.42) |
|  |  | Other^d^ |  |  |  | 0.68 (0.47, 0.995) |
| HLA genotype* *Wheat and Vegetable fats* | 24 | DQ2/DQ2^b^ | .04 | 1.15 (0.97, 1.36) | .13 |  |
|  |  | DQ2/DQ8^c^ |  | 1.08 (0.92, 1.26) |  |  |
|  |  | Other^d^ |  | 1.41 (1.17, 1.70) |  |  |
| Sex* *Meat, Rice and GF grains* | 18 | Boys | .02 | 0.85 (0.72, 0.99) | .40 |  |
|  |  | Girls |  | 1.05 (0.92, 1.19) |  |  |
| First-degree relative with CD **Fruit and Vegetables* | 24 | Yes | .046 | 0.99 (0.89, 1.09) | .58 |  |
|  |  | No |  | 1.29 (1.00, 1.66) |  |  |

^a^ Models are adjusted for HLA genotype, sex, family member with celiac disease, country, energy intake and total daily gluten intake. Only hazard ratios (HR) for statistically significant interactions are reported.
^b^ Including genotype DR3*0501/0201*DR3*0501/0201
^c^ Including genotype DR4*030X/0302*DR3*0501/0201
^d^ Including genotypes DR4*030X/0302*DR4*030X/0302, DR4*030X/0302*DR4*030X/020X, DR4*030X/0302*DR8*0401/0402, DR4*030X/0302*DR1*0101/0501, DR4*030X/0302*DR13*0102/0604, DR4*030X/0302*DR4*030X/0304, DR4*030X/0302*DR9*030X/0303, DR3*0501/0201*DR9*030X/0303.
*Abbreviations: CD = Celiac disease, GF=gluten-free*
